# P-1256. Comparing Predictiveness of Voriconazole Population Pharmacokinetic Models for Use in Model-Informed Precision Dosing in Children and Young adults

**DOI:** 10.1093/ofid/ofaf695.1447

**Published:** 2026-01-11

**Authors:** Maria-Stephanie Hughes, Laura Bio, Kevin J Downes, Anna Sharova, Jasmine Hughes, Dominic M H Tong

**Affiliations:** InsightRX, Boston, MA; Lucile Packard Children's Hospital Stanford, Palo Alto, CA; Children's Hospital of Philadelphia, Philadelphia, PA; Children's Hospital of Philadelphia, Philadelphia, PA; InsightRX, Boston, MA; InsightRX, Boston, MA

## Abstract

**Background:**

Model-informed precision dosing (MIPD) may improve voriconazole treatment in children by enabling more accurate PK assessments with earlier sampling. This study evaluates the predictiveness of voriconazole population pharmacokinetic (popPK) models in pediatric and young adult patients.

**Table 1.**
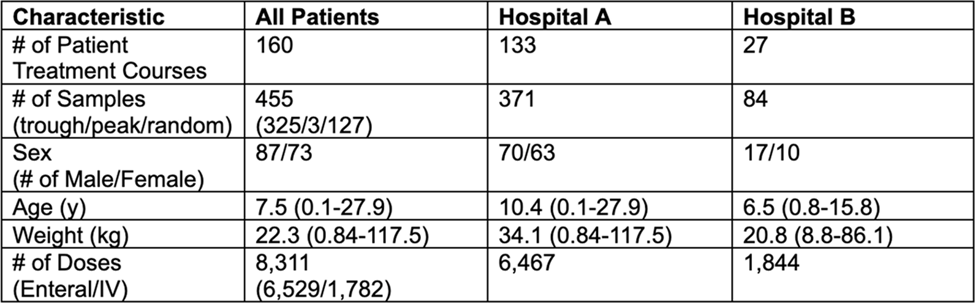
Patient, Dosing and TDM Characteristics Counts are reported when # is indicated, otherwise medians and ranges are reported. Troughs were defined as samples taken within 1 hour before dose administration, peaks were defined as samples taken within 2 hours after end of infusion, and randoms were defined as all other levels.

**Figure 1.**
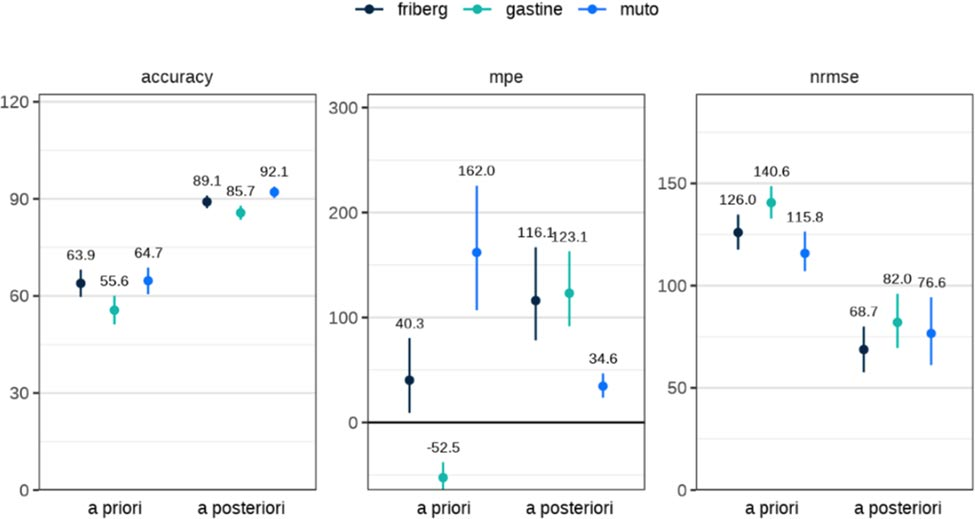
Predictive Performance of PopPK Models: All Doses Accuracy, MPE, and nRMSE are reported and summarized on all patients, regardless of dosing route, both a priori and a posteriori for all three models (Friberg, Gastine, and Muto). Numbers above each datapoint represent the median and error bars show 5th-95th percentiles calculated across 1000 bootstraps.

**Methods:**

Demographic, dose administration, and therapeutic drug monitoring sample (TDM) data on patients receiving voriconazole intravenously (IV) or enterally at two children’s hospitals were retrospectively evaluated. Patients with ≥1 voriconazole serum concentration obtained during their treatment course were included. Three published fit for purpose popPK models were assessed for predictive performance both before a sample was taken (*a priori*) and after (*a posteriori*) using Perl-speaks-NONMEM proseval. Initial concentrations were predicted using population PK parameters and patient covariates; subsequent concentrations were iteratively predicted using maximum a posteriori (MAP) Bayesian estimation based on all prior observed concentrations.Bias was measured by calculating mean percentage error (MPE), precision by normalized root mean squared error (nRMSE) and accuracy was measured and defined as a predicted level being within 2 mg/L of the measured value, or within 50% due to the low absolute concentrations measured.

**Figure 2.**
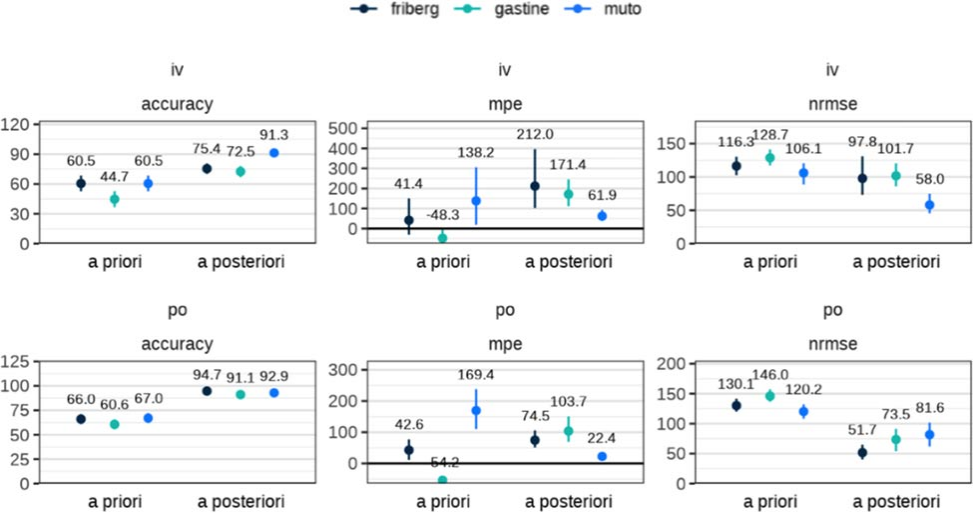
Predictive Performance of PopPK Models: IV and Enteral

Accuracy, MPE, and nRMSE are reported and summarized on all patients, split up by dosing route, both a priori and a posteriori for all three models (Friberg, Gastine, and Muto). The top row of figures are IV route and the bottom row of figures are enteral route (referred to here as PO). Numbers above each datapoint represent the median and error bars show 5th-95th percentiles calculated across 1000 bootstraps.

**Results:**

A total of 160 patient treatment courses (455 levels, predominantly troughs) were included. The average patient age was 7.5 y (range: 0.1-27.9) and average weight was 22.3 kg (range: 0.84-117.5) (Table 1). *A priori* predictions demonstrated inadequate performance across models, with accuracy ranging from 55.6-64.7%, MPE from 40.3-162.0%, and nRMSE from 115.8-140.6%. Performance improved *a posteriori*, with accuracy increasing to 85.7-92.1%, MPE decreasing to 34.6-123.1%, and nRMSE to 68.7-82.0% (Figure 1). Model accuracy was higher in enteral dosing compared to IV (Figure 2).

**Conclusion:**

We evaluated voriconazole popPK model performance for Bayesian estimation at two children’s hospitals. *A priori* predictions (using covariates alone) were poor. *A posteriori* predictions (after sampling) were better but not consistently adequate to guide dosing.

**Disclosures:**

Maria-Stephanie Hughes, PharmD, InsightRX: Employee of company|InsightRX: Stocks/Bonds (Private Company) Kevin J. Downes, MD, Paratek Pharmaceuticals, Inc.: Grant/Research Support|Veloxis Pharmaceuticals, Inc.: Grant/Research Support Jasmine Hughes, PhD, InsightRX: Employee Dominic M.H. Tong, PhD, InsightRX: Employee of company|InsightRX: Stocks/Bonds (Private Company)

